# Successful resuscitation of an elderly man with deep accidental hypothermia using portable extracorporeal circulation in the emergency department: a case report

**DOI:** 10.1186/1752-1947-2-150

**Published:** 2008-05-09

**Authors:** Simone S Cooper, Thomas J Papadimos, Jeffery A Campbell, Gregory J Cerilli, Shuab Omer, Anthony L Braida, Ali M Hassan

**Affiliations:** 1 Department of Anesthesiology, University of Toledo, College of Medicine, Toledo, OH 43614, USA; 2 Department of Perfusion, University of Toledo Medical Center, Toledo, OH 43614, USA; 3 Department of Surgery, University of Toledo, College of Medicine, Toledo, OH 43614, USA

## Abstract

**Introduction:**

Deep accidental hypothermia (body temperature below 28°C) is rare and has a high mortality rate. Successful resuscitation usually occurs in the young, but a prompt intervention using a portable extracorporeal cardiopulmonary circulation device can also provide a good outcome for older persons.

**Case presentation:**

We report the successful resuscitation of an 82-year-old male from deep accidental hypothermia using portable extracorporeal circulation in the emergency department.

**Conclusion:**

This successful resuscitation of an 82-year-old patient demonstrates that a prompt intervention by a medical team that trains together, using a mobile cardiopulmonary bypass device via a percutaneous approach, can potentially provide good outcomes for all victims of deep accidental hypothermia, both in the operating suites and the emergency department.

## Introduction

Deep accidental hypothermia (DAH), defined as a body temperature below 28°C, occurs infrequently, but has a mortality rate of 80% [1]. While there are several choices for intervention in hypothermia treatment, extracorporeal circulation is one of the most widely accepted clinical techniques for the treatment of DAH [2,3]. We report the successful resuscitation of an elderly man from DAH using portable extracorporeal circulation in an emergency department (ED).

## Case presentation

An 82-year-old man was found unresponsive on his backyard porch. His wife had gone to bed at 10 p.m. the evening before and did not realize he was missing until 6.30 a.m. the next morning. The ambient temperature was -10°C with a wind chill factor of -20°C. His past history included Alzheimer's disease, hypertension, hypothyroidism, a cerebrovascular accident in 1999 with a residual expressive aphasia, and surgery for prostate cancer. His medications included aspirin, olanzapine, alendronate sodium, and levothyroxine. He had an allergy to indomethicin.

He was flown by helicopter to the Level I trauma center. During prehospital resuscitation, he received intravenous lidocaine 100 mg, etomidate 30 mg, midazolam 5 mg, and 2 liters of warm 0.9% NaCl. As the aircraft landed, a pulse and blood pressure became unobtainable. He arrived on a backboard with a cervical collar in place, his trachea intubated, and a 20 gauge intravenous line infusing 0.9% NaCl. Cardiopulmonary resuscitation (CPR) was initiated (see Table 1). An electrocardiogram (ECG) confirmed pulseless electrical activity (PEA) with a heart rate of 31 beats per minute. His lungs were clear to auscultation bilaterally. His pupils were fixed and dilated at 4 mm, and he had a rectal temperature of 25.5°C. He was severely cyanotic to his nipple line and in both upper extremities. A chest roentgenogram revealed the tracheal tube was in the proper position. He was given atropine 1 mg intravenously for his PEA, this was followed by 1 mg epinephrine intravenously. The patient converted to sinus rhythm, but within 90 seconds he went into ventricular tachycardia and subsequently into ventricular fibrillation; CPR was continued. Ventricular defibrillation attempts were withheld because of the critically low temperature of the patient. Two additional 16 gauge intravenous catheters and a left subclavian catheter were placed and warm 0.9% NaCl was instilled. A nasogastric tube and bilateral chest tubes were also placed to instill warm fluid. Laboratory examination revealed sodium 142 meq/liter, potassium 3.3 meq/liter, chloride 113 meq/liter, HCO_3 _19 mmol/liter, glucose 128 g/dl, lipase 28 U/liter, calcium 8.5 mg/dl, total bilirubin 0.9 mg/dl, alkaline phosphatase 57 U/liter, aspartate transaminase 35 U/liter, total protein 5.5 g/dl, albumin 2.8 g/dl, and lactate 3.0 mmol/liter. Arterial blood gas (ABG) was not drawn at this time. Thyroid stimulating hormone (TSH) and T4 levels were drawn (TSH was 9.02 micro-IU/ml (normal 0.10 to 5.0 micro-IU/ml) and his T4 was 1.6 mcg/dl (normal 4.5 to 13.2 mcg/dl), but the results were not available until after resuscitation). His temperature did not change after nearly 60 minutes of conventional warming therapies that included warm fluid instillation (intravenous, gastric, and intrapleural), use of a warming blanket, and CPR in a trauma room that was 27°C. The perfusion service was consulted and it was determined that the patient would benefit from rewarming by extracorporeal circulation.

**Table 1:** Temporal sequence of resuscitation events

Time	Patient status/Event
0715 – 0800 Pre-Hospital	Patient was unresponsive with absent pedal pulses but palpable radial pulses with delayed capillary refill. A 20 ga. IV line was established and patient was intubated. Vitals were bradycardia with HR 33/minute, BP of 86/51 mm Hg, and tympanic temperature of 25.5°C. Warm IV fluids were given and hot packs were applied to groins and axillae.
0800 Arrival to Hospital	Patient arrived to medical center with no palpable pulses. Monitor showed PEA with bradycardia of HR 31/minute. CPR was started. Atropine 0.5 mg IV was administered followed by 1 mg epinephrine IV. Tympanic temperature was 25.5°C.
0808	Patient converted to sinus rhythm with HR 66/minute, but shortly thereafter went into pulseless V-tach. An amiodarone bolus of 300 mg was administered IV followed by 1 gm calcium IV. The patient went into V-fib and CPR resumed.
0812	Central line placed along with an additional 16 ga. IV line. Warm IV fluids were administered.
0815 – 0825	A gastric tube and bilateral chest tubes were placed. Warm gastric and pleural irrigation was initiated.
0845	Patient remained in V-fib arrest receiving CPR. Temperature remained 25.6°C despite conventional warming therapies of warm fluid instillation (IV, gastric, intrapleural), warm humidified oxygen, and warming blankets. The decision was made for extracorporeal rewarming and resuscitation.
0845 – 0900	Patient was heparinized, CPS unit was assembled and primed, and arterial and venous cannulas were placed for fem-fem CPS.
0900	Patient was placed on fem-fem CPS and CPR was stopped. Patient remained in V-fib arrest. CPS unit temperature monitoring confirmed patient blood temperature of 25.5°C.
0900 – 1100	Patient was slowly rewarmed on CPS and electrolytes and acid base status were normalized. Patient remained in V-fib arrest. An arterial line was placed which showed a systolic BP of 55 mm Hg. Vasopressin was started.
1102	Patient temperature was 34.9°C and defibrillation was attempted. Initial defibrillation at 200 J converted rhythm from V-fib to V-tach, and a subsequent defibrillation of 300 J converted rhythm to SVT. A bolus of 300 mg amiodarone IV was administered followed by 1 gm calcium chloride IV.
1112	Patient reverted into V-tach. Two defibrillation attempts of 360 J converted rhythm into sinus tachycardia and HR 102/minute. Patient temperature was 35.5°C.
1200	Patient temperature was 37°C after 180 minutes of extracorporeal rewarming.
1230	Patient was transported to OR for weaning of CPS and decannulation under direct vision. Patient was in sinus rhythm, HR 87/minute.
1330	Patient was successfully weaned from CPS after 270 minutes of bypass with BP 140/60, HR 80/minute, in sinus rhythm, and temperature of 37°C.

IV: intravenous; HR: Heart Rate; BP: Blood Pressure; J: joules; PEA: Pulseless Electrical Activity; CPR: Cardiopulmonary Resuscitation; V-Tach: Ventricular Tachycardia; V-Fib: Ventricular fibrillation; A-V: Arterio-Venous; CPS: Cardiopulmonary Support; Fem: femoral; SVT: Supraventricular Tachycardia

A heparin bolus of 200 U/kg of body weight was given to target an activated clotting time of 300 seconds. Cannulation of the right common femoral artery and vein, using 20 French percutaneous femoral arterial and venous cannulas (Edwards Lifesciences, Irvine, CA) was achieved via surgical cutdown. The patient was placed on emergency percutaneous veno-arterial femoral-femoral bypass in the ED using a self-contained, portable cardiopulmonary bypass (CPB) support system (PBS Portable Bypass System, Medtronic, Inc., Minneapolis, MN) consisting of a portable centrifugal blood pump console with an external drive motor and heater unit. The pre-assembled perfusion circuit consisted of a Biomedicus centrifugal pump (Medtronic, Inc., Minneapolis, MN). CPB was initiated within 10 minutes of the decision for its use and 58 minutes after the arrival of the patient to the ED; the patient's core temperature was 25°C.

**Figure 1:**
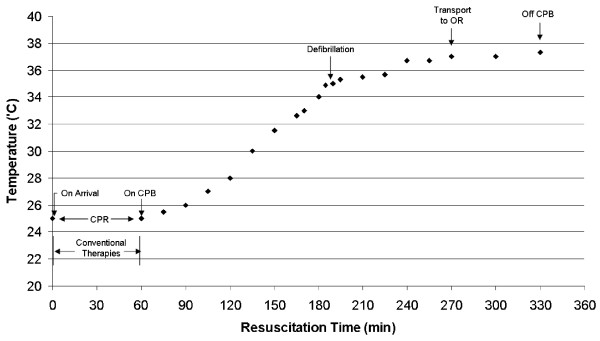
Temporal events related to the rewarming of the patient.

An initial venous blood gas drawn from the perfusion circuit revealed a pH of 7.16, pCO_2 _32 mmHg, pO_2 _60 mmHg (FIO_2 _1.0), sodium 148 meq/liter, potassium 2.8 meq/liter, calcium 1.04 meq/liter, HCO_3 _11 mmol/liter, a base excess of -15.0 mmol/liter, and a hematocrit level of 14.8%. The patient was stabilized at this temperature for 15 to 20 minutes prior to commencing core rewarming to provide the opportunity for reintroducing generalized perfusion and gas exchange prior to actively rewarming. Potassium was repleted, and the mean arterial pressure was maintained at 66 mmHg. Defibrillation attempts were withheld until the core temperature reached 35°C. Successful defibrillation was achieved after 120 minutes of core rewarming, at a core temperature of 34.9°C (Figure 1). The patient was then transferred to the operating room for decannulation and repair of the femoral vessels under direct vision. His hemodynamic parameters were stabilized with a resultant ABG of pH 7.42, PaCO_2 _38 mmHg, PaO_2 _82 mmHg, and HCO_3 _24 mmol/liter, base excess 0 mmol/liter on 0.6 FIO_2_. He was transferred to the intensive care unit (ICU).

His hemodynamic support necessitated the use of large amounts of fluids and blood products secondary to a severe coagulopathy of hypothermia and subsequent vasodilation due to rewarming. He received 12 liters of crystalloid, 15 U of packed red blood cells, 20 U of platelets, and 2 U of fresh frozen plasma during the first 7 hours of his hospitalization. Twelve hours after admission prothrombin time was 16.1 seconds, partial promboblastin time was 32.6 seconds, and international normalized ratio was 1.23.

It was determined that he had sustained a myocardial infarction during his perihypothermic time interval (creatinine kinase 988 U/liter, creatinine kinase antibodies 109.6 ng/ml, CK-MB index 11.1 ng/ml, myoglobin 2323 ng/ml and troponin I 117.65 ng/ml). His ECG demonstrated occasional premature ventricular complexes, low-voltage QRS, non-specific ST and T wave abnormality, and a prolonged QT interval. However, transesophageal echocardiography 72 hours after admission revealed a normal global ventricular function, normal left ventricle, normal aortic, mitral and pulmonary valves, normal left and right atrial size, normal ventricular thickness, mild tricuspid regurgitation, mildly elevated right-sided pressures, and a small anterior pericardial effusion.

Five days after admission he underwent placement of a tracheostomy and gastric feeding tube. His mental status recovered to its premorbid state 10 days after admission, but he was physically debilitated. Three weeks after admission he was discharged to a long-term acute care hospital. Subsequently he was transferred to a nursing home where he was still residing 6 months after hospital discharge.

## Discussion

Every year in the US, 4 in every 1,000,000 people die as a result of hypothermia. In the period 1999 to 2002, 4607 death certificates had hypothermia-related diagnosis and/or injury as the underlying cause of death [4]. Hypothermia has been defined as a core body temperature below 35°C with three classifications: mild 32.2 to 35°C, moderate 28 to 32.2°C, and deep below 28°C [5]. Surviving DAH is rare [5,6] and is closely related to youth [7]. Here we report not only the oldest survivor of DAH, but also demonstrate that a self-contained portable CPB support system, using percutaneous cardiopulmonary support (CPS), can be used in the ED to facilitate the survival of such a patient. In addition, we must acknowledge that this patient's subtherapeutic T4 may have also contributed to his hypothermia (although TSH and T4 levels were not correct until after discontinuation of CPS).

Multiple factors contributed to this successful clinical outcome. Walpoth et al. [7] have enumerated five factors needed for a favorable outcome in this scenario. The first factor is youth (which is not applicable here, but the other factors were relevant). Second, the patient had deep hypothermia. Third, although the patient had Alzheimer's disease, no asphyxia or hypoxic brain damage occurred during the event. Fourth, our trauma services were well organized; the emergency, surgery, and anesthesiology departments engage in joint teaching, training, research, and administrative activities. Fifth, we used an effective rewarming technique applied properly and in a timely fashion. While CPR did proceed for nearly 60 minutes before application of CPB, there are instances of longer resuscitation times before the start of a successful resuscitation with extracorporeal circulation [8].

CPS using a compact mobile CPB machine is a modality that originated in the late 1980s, primarily for use in the cardiac catheterization laboratory for assisted angioplasty and resuscitation [9]. This became possible after the development of thin-walled cannulas that allowed for percutaneous femoral cannulation using the Seldinger technique. This technique provided adequate venous drainage and systemic flows. The CPB circuit is pre-assembled, which allows for quick set-up and initiation. Implementation of CPS support can be performed during CPR, it is rapid, and does not require a cardiothoracic surgeon, mobilization of a cardiothoracic team, or transfer to a cardiac operating room. This modality has evolved to provide cardiopulmonary support and resuscitation in other hospital settings, such as the ICU and ED, and in other clinical situations to include cardiogenic shock, witnessed cardiac arrest, massive pulmonary embolism, near drowning, drug overdose, and severe hypothermia [10].

Femoral vein-femoral artery CPB was chosen as the method of intervention. However, good results have been achieved with veno-venous hemofiltration, peritoneal dialysis, and hemodialysis [11,12]. Femoral-femoral vascular cannulation for CPB was selected to avoid complications associated with sternotomy, such as bleeding secondary to hypothermic coagulopathy, pain control and wound infection [13]. Furthermore, since the equipment and expertise were available to the ED, the decision was made not to transport a critically ill patient undergoing CPR to a cardiothoracic operating suite.

Using the femoral route is not without complications. Such access could contribute to ventricular dilation and cause pulmonary edema in addition to being associated with inadequate venous return, distal limb ischemia, pseudo-aneurysm, retroperitoneal bleeding, and sepsis [14,15]. However, by forgoing sternotomy, we feel we provided better rewarming and hemodynamic support, and a less-invasive procedure in a safer, more prompt fashion.

## Conclusion

While most successful resuscitations from DAH occur in the young, the successful resuscitation of an 82-year-old patient demonstrates that a prompt intervention by a medical team that trains together, using a mobile CPB device via a percutaneous approach, can potentially provide good outcomes for all victims of DAH, both in the operating suites and the ED.

## Abbreviations

ABG: arterial blood gas; CPB: cardiopulmonary bypass; CPR: cardiopulmonary resuscitation; CPS: cardiopulmonary support; DAH: deep accidental hypothermia; ECG: electrocardiogram; ED: emergency department; ICU: intensive care unit; PEA: pulseless electrical activity; TSH: thyroid stimulating hormone.

## Competing interests

The authors declare that they have no competing interests.

## Authors' contributions

SSC, TJP, GJC, SO, ALB, and AMH wrote or contributed to the writing of the manuscript, except for the cardiopulmonary perfusion section, which was written by JAC. All authors read and approved the final manuscript.

## Consent

Written informed consent was obtained from the patient's next-of-kin for publication of this case report and any accompanying images. A copy of the written consent is available for review by the Editor-in-Chief of this journal.
